# Advanced Dentistry Biomaterials Containing Graphene Oxide

**DOI:** 10.3390/polym16121743

**Published:** 2024-06-19

**Authors:** Doina Prodan, Marioara Moldovan, Stanca Cuc, Codruţa Sarosi, Ioan Petean, Miuța Filip, Rahela Carpa, Rami Doukeh, Ioana-Codruta Mirica

**Affiliations:** 1 Raluca Ripan Institute for Research in Chemistry, Babeș-Bolyai University, 30 Fantanele Str., 400294 Cluj-Napoca, Romania; doina.prodan@ubbcluj.ro (D.P.); marioara.moldovan@ubbcluj.ro (M.M.); stanca.boboia@ubbcluj.ro (S.C.); miuta.filip@ubbcluj.ro (M.F.); 2 Faculty of Chemistry and Chemical Engineering, Babes-Bolyai University, 11 Arany János Str., 400028 Cluj-Napoca, Romania; ioan.petean@ubbcluj.ro; 3 Department of Molecular Biology and Biotechnology, Faculty of Biology and Geology, Babes Bolyai University, 1 M. Kogalniceanu Street, 400084 Cluj-Napoca, Romania; rahela.carpa@ubbcluj.ro; 4 Department of Well Drilling, Extraction and Transport of Hydrocarbons, Faculty of Petroleum Refining and Petrochemistry, Petroleum-Gas University of Ploiesti, 39 Bucharest Blvd, 100680 Ploiesti, Romania; 5 Department of Oral Health, Iuliu Hatieganu University of Medicine and Pharmacy, 15 Victor Babeș Str., 400347 Cluj-Napoca, Romania; mirica.ioana@umfcluj.ro

**Keywords:** posterior restoration, cement, graphene oxide, hydroxyapatite, antibacterial properties

## Abstract

The aim of this study was to obtain three experimental resin-based cements containing GO and HA-Ag for posterior restorations. The samples (S0, S1, and S2) shared the same polymer matrix (BisGMA, TEGDMA) and powder mixture (bioglass (La_2_O_3_ and Sr-Zr), quartz, GO, and HA-Ag), with different percentages of graphene oxide (0%, 0.1%, 0.2% GO) and silver-doped hydroxyapatite (10%, 9.9%, 9.8% HA-Ag). The physical–chemical properties (water absorption, degree of conversion), mechanical properties (DTS, CS, FS), structural properties (SEM, AFM), and antibacterial properties (*Staphylococcus aureus*, *Enterococcus faecalis*, *Streptococcus mutans*, *Porphyromonas gingivalis*, and *Escherichia coli*) were investigated. The results showed that the mechanical properties, except for the diametral tensile test, increased with the rise in the %GO. After 28 days, water absorption increased with the rise in the %GO. The surface structure of the samples did not show major changes after water absorption for 28 days. The antibacterial effects varied depending on the samples and bacterial strains tested. After increasing the %GO and decreasing the %HA-Ag, we observed a more pronounced antibacterial effect. The presence of GO, even in very small percentages, improved the properties of the tested experimental cements.

## 1. Introduction

Resin-based composite materials are the first choice for dental restorations. It is necessary that the restorative dental composites make a connection between their artificial structure and the natural dental structure to restore the integrity of the oral tissues [1]. In addition to their filling and sealing role, the formulation of restorative materials must take into account the preservation of the remaining dental structure and the prevention of bacterial invasion after restoration.

Graphene, as a 2D material, can be used for protective coatings, as a filler in compo-sites, or as an additive in fluids. It can improve the mechanical characteristics of composite materials. Incorporating 2D materials as fillers in composites provides material uniformity, interface connections, and design modifications [2].

Dentin is composed of hydroxyapatite, collagen, and water. Hydroxyapatite, in the presence of acidic oral fluids, can be easily removed, resulting dental demineralization. Dental erosion and caries are the result of dentin demineralization. Therefore, there is a special interest for the inclusion in the composition of dental materials of some agents with a remineralization role and with a potential antibacterial effect. The demineralization process can be prevented or stopped from the irreversible evolution of the loss of dental structure by preventing the formation of the bacterial biofilm with the help of an antibacterial agent and by introducing a remineralization agent. Combining graphene oxide with hydroxyapatite, good biomimetic mineralization and osteogenic differentiation is obtained [3]. A sealing of the dentinal tubules could reduce the demineralization and permeability of the dentin, reducing its hypersensitivity. In one study, the combination of GO with different nanoparticles of silver (Ag), zinc (Zn), calcium fluoride (CaF_2_) and tricalcium phosphate (Ca_3_(PO_4_)_2_) was investigated to evaluate their antibacterial and anti-demineralizer effects. It was found that these combinations were able to inhibit cariogenic bacteria, prevent demineralization, and have a remineralizing effect [4].

In some studies [5,6], regarding the biocompatibility of experimental adhesives, it was demonstrated that the addition of functionalized graphene particles had a beneficial influence on the biocompatibility of experimental adhesives.

Also, the investigation of water sorption is very important, because it can negatively or positively influence composite restorative materials. A reduced humidity can be beneficial because it can decrease the polymerization shrinkage of the material by relaxing the stress caused by the polymerization. However, a significant hydroscopic expansion can lead to a hydrolytic degradation of the restoration due to the infiltration phenomenon, reducing its durability [7].

The addition of graphene as a filler in dental composites leads to a good mechanical performance, while the addition of hydroxyapatite provides a low mechanical stability [6]. Xie et al. found that the addition of graphene improved the mechanical properties and biocompatibility of the dental composite [8]. A combination of the two (GO and HA) could lead both to improvements in the mechanical properties and to the realization of the remineralization process.

It has been demonstrated that the addition of alumina, hydroxyapatite, silica, or zirconium nanoparticles as fillers to the organic matrix increases the resistance to the masticatory stress and the wear resistance during mastication [9,10,11].

Data from the literature [12] reports that the strontium ions, originating from bioactive glass containing Sr, can inhibit osteoclastic resorption, thus leading to the stimulation of bone regeneration both in vitro and in vivo. Therefore, the glass with Sr is indicated to be included in the composition of the dental cements as a helper of the bone substitution and to ensure bioactivity and anticariogenic potential by releasing of the Sr ions. The commercial materials include Fuji ionomer cement IX (GC Europe, Leuven, Belgium), GuttaFlow Bioseal (Coltene, Langenau, Germany), and other Sr-based bioactive glass composites used as obturation materials in the endodontic treatment [12].

As in the case of the strontium ions, lanthanum ions (La^3+^) from lanthanum glass can induce anti-inflammatory and bactericidal properties. They have a healing impact, and they stimulate the bioactivity and biocompatibility of the material [13]. The incorporation of antibacterial agents, such as silver or some antibiotics, improves the biocompatibility of composites by ensuring antibacterial protection.

The strength of resin-based dental nanomaterials makes them suitable for both anterior and posterior restorations [14]. The materials used in dental restorations (resins, cements, adhesives) may include graphene nano-sheets to avoid adhesion and bacterial growth. Also, the ability of the GO to attach to different types of biomolecules makes them useful in a series of biological applications [15]. The functionalization of graphene, for example with silane, leads to a change in the properties of graphene oxide, resulting in deeper interfacial interactions [16].

Considering the previous results [5,17,18,19], we try to expand the research area and obtain or improve upon other types of dental materials that include different mixtures and concentrations of the powders obtained by us to increase the durability of dental restorations.

The null hypothesis is that %wt GO/%wt HA in the composition of the restorative cements does not influence their properties.

The novelty of this study is the identification of the optimal content of GO and HA-Ag in three experimental composite cements for posterior dental restorations. This research investigates how these powders, as well as changes in their proportions, affect the cements’ degree of conversion, mechanical properties, water sorption, structural characteristics, and antibacterial activity.

## 2. Materials and Methods

### 2.1. Materials

For this study, 3 experimental composite pastes were formulated with an organic matrix based on Bis-GMA/TEGDMA and experimental inorganic fillers (Table 1). The pastes had a similar composition. The difference was in the percentage of graphene oxide (GO) and hydroxyapatite with silver (HA-Ag) added.

The La-Zr nanofiller [17] was obtained using the sol-gel method, and the glass with Sr-Zr [18] was obtained via the classic melting process.

The GO powder was obtained using the Hummer method [5,19] which was carried out according to a previous study, starting from graphite flakes (10 mesh) and NaNO_3_ mixed in a ratio of 2:1. The mixture of powders was slowly added over concentrated H_2_SO_4_ (230 mL) while stirring at a temperature below 5 °C. KMnO_4_ was added slowly (3:1 to the initial amount of graphite flakes), and the mixture was stirred for 30 min at a temperature below 15 °C, after which the temperature was gradually increased to 35 °C for another 30 min. Distilled water (460 mL) was added dropwise, increasing the temperature to 95 °C, after which the rest of the water (1400 mL) was added, followed by the dropwise addition of 100 mL of hydrogen peroxide (H_2_O_2_) to complete the reaction. After filtration, the mixture was washed several times with 5% HCl and then with distilled water. GO powder was obtained via lyophilization of the precipitate.

Hydroxyapatite (HA) was obtained using the wet chemical method (precipitation technique), and HA with silver (HA-Ag) was obtained by adding silver nitrate (AgNO_3_) to the HA gel [5].

The powders were obtained at the Polymeric Composites Laboratory of UBB-ICCRR. The silanization of the powders was carried out with silane A-174 (3-methacryloyloxypropyl trimethoxy silane) hydrolyzed in acidified alcoholic solution [17]. Regarding the photochemical polymerization system, 0.5% camphorquinone and 1% dimethyl aminoethyl methacrylate (CQ, DMAEM) were related to the amount of liquid (organic matrix) and incorporated into it. The composite cements were obtained in the form of a paste by incorporating the inorganic filler in the organic matrix, after which they were stored in dark containers. The samples specific for each test were obtained via photochemical polymerization of the materials in Teflon molds with the help of a dental LED photopolymerization lamp (Woodpecker Medical Instrument Co., Guilin, China).

### 2.2. Methods

The size of the specimens used for the mechanical tests and water absorption are shown in the respective Section 2.2.2 and Section 2.2.3 and were made according to the indications of ISO 4049/2000 [20]. For the microstructural analyses, water absorption samples were used (initially and at the end of the storage period in distilled water). The recordings for the conversion test were performed on pastes and on samples in the form of a disc (hardened paste) with dimensions of 5/1 ± 0.2 mm in diameter/thickness, respectively. Similar samples (5/1 ± 0.2 mm) were also made for the antibacterial test.

#### 2.2.1. Degree of Conversion (DC)

The DC of the cured liquid sample (polymeric matrix) and of the cement samples was determined immediately and 24 h after polymerization.

The conversion was evaluated by determining the residual C=C unsaturated double bonds that were left unreacted at the end of the polymerization. The recording of the spectra of the organic matrix (liquid and cured) and of the composites (paste and cured) after one minute and after 24 h, respectively, was carried out using an FTIR spectrophotometer (Jasco FTIR-610) equipped with an ATR attachment (attenuated total reflectance) with a horizontal ZnSe crystal (Jasco PRO400S). For the quantitative determination of the unreacted methacrylate groups, the absorption band from 1635–1640 cm^−1^ corresponding to the valence vibrations of the C=C double bonds in the methacrylic groups (Ameth) was used. As an internal standard, for the phenyl group (Aarom), the absorption band at 1605–1610 cm^−1^ was used.

The degree of conversion was calculated using Formula (1):

conv% = {1 − [Amet/Aarom]copolimer/[Amet/Aarom]monomer} × 100(1)

where A is the absorbance intensity for the Amet—the C=C double bond in the methacrylic group of the monomer and polymer, respectively, and Aarom is the C-C bond in the aromatic cycle.

#### 2.2.2. Mechanical Testing

Ten samples were obtained from each experimental cement for the mechanical properties testing. The cement pastes were inserted into Teflon molds and light cured with an LED.E dental lamp (Guilin-Wood-pecker Medical Instruments Co., Guilin, Guangxi, China) at a 470 nm wavelength. Mechanical strength tests were performed using the Universal Testing Machine (LF Plus, LLOYD, Instrument, Ametek Inc, Bognor Regis, West Sussex, UK) and Nexygen Plus 3.0 software. The cell movement speed of flexural strength was 0.5 mm/min, and for compression and diametral tensile, it was 1 mm/min.

For the compression test (CS), the cylindrical specimens with dimensions of 6 mm height × 3 mm in diameter, were cured on each side for 20 s. After this step, the samples were dried for 24 h at room temperature, after which they were measured using an electronic scale. All the samples were previously immersed in water in a thermostatic bath at 37 °C for 24 h. To evaluate the diametral tensile strength (DTS), samples with cylindrical shapes (3 mm thick and 6 mm in diameter) were prepared. The samples made for the flexural strength test (FS) had a rectangular shape and dimensions of 25 mm long, 2 mm high, and 2 mm wide. The sample curing was carried out in 5 selected areas for 20 s on both sides. The samples were subjected to a three-point bending test. The Young’s modulus (YM) for bending was determined by the slope of the linear part of the force–deflection diagram. The determinations were made according to ISO 4049/2000 [20], with calculations in MPa.

#### 2.2.3. Water Sorption (WS)

The disc-shaped specimens (7.5 radius × 1 mm thickness) for the water absorption test (N = 10) were obtained according to ISO 4049 [20]. The curing was performed in four different points, for 20 s for each point, using an LED.E dental lamp (Guilin Woodpecker Medical Instruments Co., Guilin, Guangxi, China). After drying for 24 h in a desiccator, the samples were weighted to a constant mass (m0). The samples were immersed individually in 20 mL of distilled water in containers for 28 days at 37 °C (±2). After 24 h, each sample was removed from the water, air-dried for 20 s, and weighted (m1). The subsequent drying of the samples was carried out in a desiccator until a constant mass (m2) was obtained. The investigation period was 28 days, and the samples were weighted on days 1, 2, 3, 7, 10, 14, 21, and 28. Equation (2) was used to calculate WS in [μg/mm^3^]. The results for water sorption of the samples [μg/mm^3^] were calculated using Equation (2):

WS = (m1 − m2/V)(2)

where m1 = the mass weight after 20 s of drying; m2 = the final mass weight; and V = the volume of the samples.

The standard limit for the water absorption of restorative materials is ≤40 μg/mm^3^ [20].

#### 2.2.4. Scanning Electron Microscopy (SEM)

A structural investigation of the surfaces of the cement samples before and after the WS test was carried out using a scanning electronic microscope (SEM-Inspect S, FEI Company, Hillsboro, OR, USA) at a magnification of ×2000 (×5000 the small square). Images were obtained in different areas in order to observe the potential damage of the sample surfaces after 28 days of storage in water compared to the images captured on their surface before storage in water.

#### 2.2.5. Atomic Force Microscope (AFM)

To perform the atomic force microscopy (AFM) test, we used a scanning probe microscope, JSPM 4210, produced by JEOL Co., Tokyo, Japan. The surface of the cement samples before and after the water absorption test was probed with NSC 15 cantilevers produced by MikroMasch Co., Tallinn, Estonia, having a resonant frequency of 325 kHz and a force constant of 40 N/m. The scanning rate was in the range of 1–3 Hz depending on the scan size and sample corrugation. Topographic images were processed using WinSPM 2.0 Processing soft produced by JEOL Co., Tokyo, Japan, and the surface roughness parameters Ra and Rq were measured for the sample’s fine microstructure (scanning areas of 20 µm × 20 µm and 10 µm × 10 µm) and for the ultrastructural details at a scan size of about 5 µm × 5 µm and 2.5 µm × 2.5 µm (scanning areas of 20 µm × 20 µm), respectively, and for the ultra-structural details, at scan size of about 5 µm × 5 µm.

#### 2.2.6. Antibacterial Test

*Test microorganisms*. For the antibacterial testing of samples S0, S1, and S2, the following test microorganisms were used: *Staphylococcus aureus* ATCC 25923, *Enterococcus faecalis* ATCC 29212, *Streptococcus mutans* ATCC 25175, *Porphyromonas gingivalis* ATCC 33277, and *Escherichia coli* ATCC 25922 from the collection of the Microbiology Laboratory, Faculty of Biology and Geology, UBB, Cluj.

**Figure 1:**
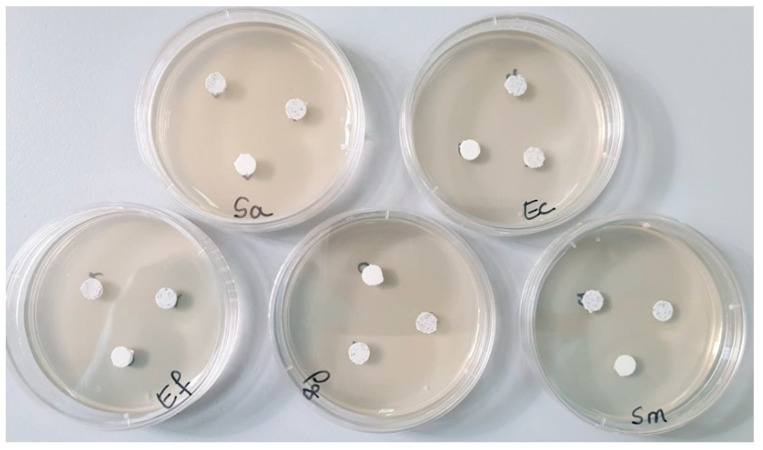
The bacterial strains studied and the samples (S0, S1, and S2) tested (Sa—*Staphylococcus aureus*, Ec—*Escherichia coli*, Ef—*Enterococcus faecalis*, Pg—*Porphyromonas gingivalis*, Sm—*Streptococcus mutans*).

*Materials*, *methods, and used equipment*. Each bacterial strain was grown for 24 h on Nutrient Agar medium [21]. Then, a dilution of 0.5 McFarland was made from each strain in sterile physiological serum. From these dilutions, each Petri dish was inoculated with the help of a sterile swab soaked in the microbial suspension of 0.5 McFarland spreading over the entire surface of the solid culture medium (Mueller Hinton-Oxoid, specific for antimicrobial testing). Then, in each Petri dish containing the culture medium inoculated with the bacteria to be tested, the 3 samples (marked S0, S1, S2) were applied (Figure 1). Incubation was performed for 48 h at 25 °C. The reading was performed by measuring the diameter of the inhibition zone with a ruler; the larger the inhibition zone diameter was, the greater the sensitivity of the bacteria to the respective samples [22].

#### 2.2.7. Statistical Analysis

A one-way ANOVA test was used for post-hoc comparisons between the sample groups, along with a Tukey test. The significance level was set to α = 0.05. For the statistical analysis, the Origin2019b graphing and analysis software (9.65) (OriginLab, Northampton, MA, USA) was used.

## 3. Results

The degree of the composite’s cements conversion provides information regarding whether the polymerization proceeded correctly.

### 3.1. Degree of Conversion (DC%)

**Figure 2:**
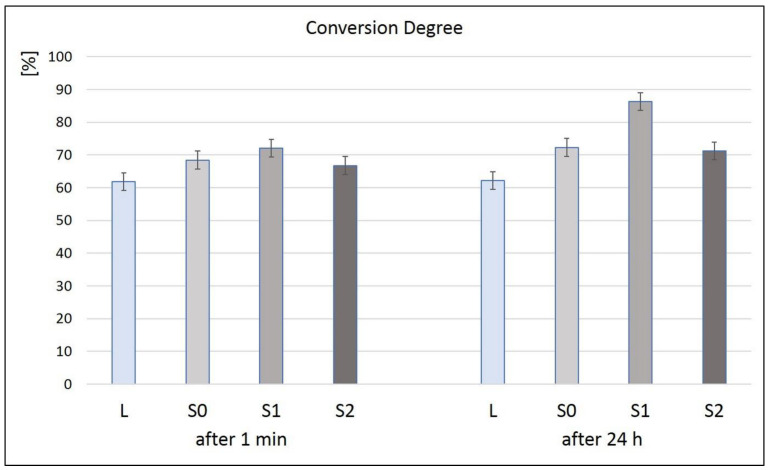
The degree of conversion of the liquid sample and of the cement samples immediately cured and 24 h after polymerization.

From Figure 2, it can be seen that the lowest degree of conversion (CD%), both immediately after polymerization and 24 h after polymerization (61.8% and 62.1%), was recorded in the case of sample L (the organic matrix sample). The highest CD%, both immediately after polymerization and at 24 h after polymerization (72% and 86.2%, respectively), was recorded by sample S1. After 24 h, the CD% of all the samples increased, which meant that polymerization continued over time. After 24 h, the CD% of all the samples with inorganic filler (S0–S2) was over 70%.

Statistically comparing the obtained results, significant differences were obtained both in the batch analyzed after 1 min of polymerization (between L–SO and L–S1) and in the batch 24 h after the polymerization reaction (among all four samples analyzed, outside SO and S2). If we compare the DC% as a function of the analysis over time, there are significant differences for S1 after 1 min and S1 after 24 h, but there are no differences for the other three samples analyzed.

### 3.2. Mechanical Testing

Table 2 shows the average values of flexural strength (FS), compression (CS), diametral tensile strength (DTS), and Young’s modulus (YM) of cement samples with the standard deviation (SD).

**Figure 3:**
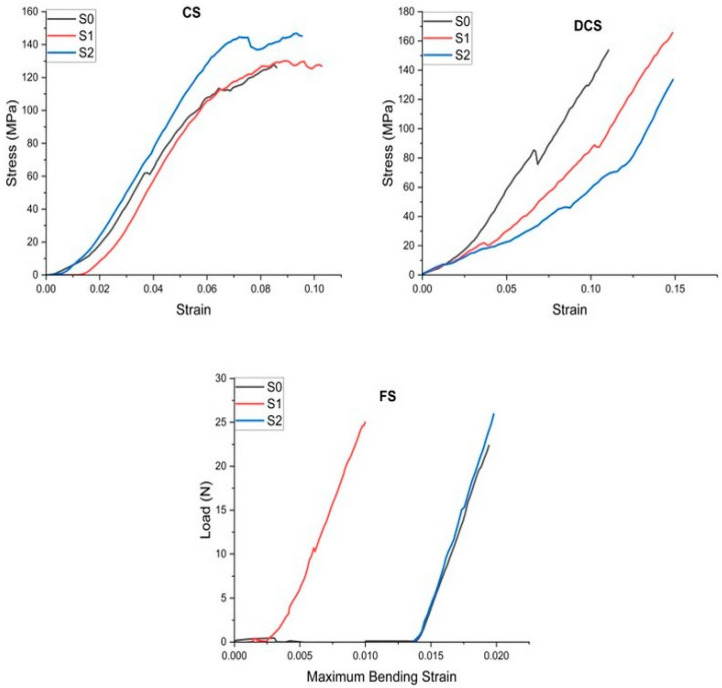
Stress–strain curves for the mechanical tests.

Figure 3 shows that the addition of graphene oxide to the S1–S2 samples influenced the mechanical properties of both cements differently. The highest resistances to CS, FS, and YM were obtained for cement sample S2, but the lowest DTS value was recorded for sample S2. From the collected data, it can be seen that the CS, FS, and MY increase with the growth in the percentage of added graphene oxide. With the addition of 0.1% GO (sample S1), it is observed that the DTS increases, but with the addition of 0.2% GO, the DTS decreases (sample S2). The cement sample without the addition of GO (S0) had a lower CS and FS compared to cement samples S1 (0.1% GO) and S2 (0.2% GO). The DTS was higher than sample S2 but lower than sample S1. The modulus of elasticity, however, was higher than that of sample S1 but lower than that of sample S2.

### 3.3. Water Sorption (WS)

**Figure 4:**
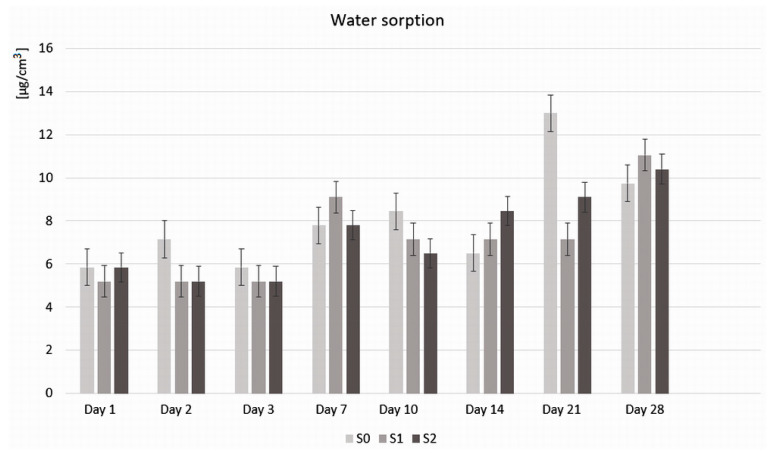
Water absorption of sample S0 (without GO) and samples S1 and S2 (with 0.1% and 2% GO, respectively) after 1, 2, 3, 7, 10, 14, 21, and 28 days.

From Figure 4, it can be seen that, after 24 h, cement sample S0 (without GO) and cement sample S2 (with GO 2%) absorbed 5.8 μg/cm^3^ of water, while sample S1 (with GO 1%) absorbed 5.1 μg/cm^3^ of water. At the end of the investigation period, it can be seen that the largest amount of water was absorbed by cement sample S1 (11.0 μg/cm^3^), followed by sample S2 (10.3 μg/cm^3^) and then by sample S0 (9.7 μg /cm^3^). A non-uniform absorption of the S0 sample was also observed throughout the investigation period, with increases and decreases from one day to the next, with the highest increase on day 21 (12.9 μg/cm^3^). The GO samples (S1 and S2) absorbed the same amount of water on days 2 and 3 (5.1 μg/cm^3^), less than the S0 sample. On day 7, samples S0 and S2 absorbed the same amount of water (7.7 μg/cm^3^), while sample S1 absorbed more (9.0 μg/cm^3^). After this increase, the amount of water absorbed by sample S1 decreased (7.1 μg/cm^3^) and remained constant in the interval of 10–14–21 days. After 14 days, sample S2 had an absorption value (8.4 μg/cm^3^) similar to that of sample S0 on day 10. On day 21, the water absorption of sample S2 increased (9.0 μg/cm^3^), similar to that of sample S1 on day 7.

Comparing the water absorption evolution of the three investigated samples during the 28 days, there were no statistically significant differences between the samples (*p* = 67,995). Comparing the evolution of each sample during the 28 days, there were differences for each sample, between the first 3 days and the following 3 and between days 14 and 28.

### 3.4. Scanning Electron Microscopy (SEM)

**Figure 5:**
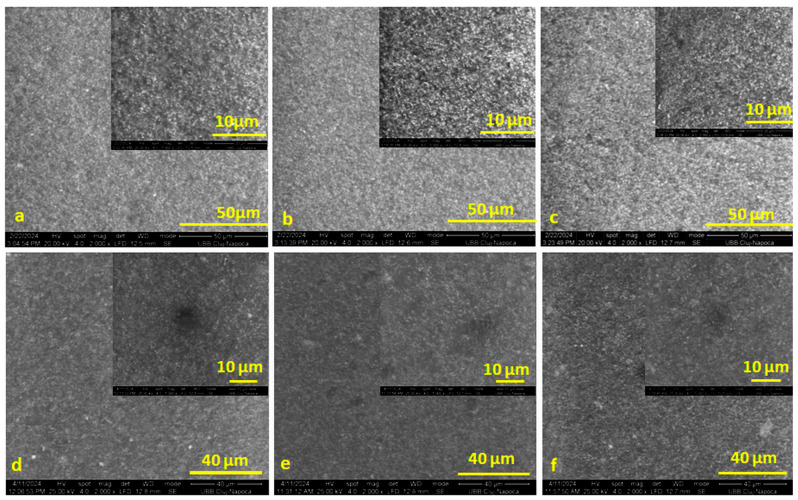
SEM images on the surface of samples S0 (**a**,**d**), S1 (**b**,**e**), and S2 (**c**,**f**) before the water absorption test (**a**–**c**) and after 28 days of storage in water (**d**–**f**).

From the images captured on the initial surface of the samples (Figure 5a–c), a uniform structure of the surfaces can be observed, which was very similar for all three samples. 

After the absorption test (Figure 5d–f), no significant difference could be observed between the surface structures of the samples. Smoother surfaces could be observed on each sample due to the accumulation of water in the pores and small processing defects.

The surfaces were sprinkled with small round areas that were darker in color; most likely areas where water entered the microbubbles with embedded air when mixing the cement paste components. The water managed to penetrate the microbubbles, removing the particles embedded in the resin film from their surface without penetrating too deeply. The most uniform surface (before and after absorption) was that of cement S1.

### 3.5. Atomic Force Microscope (AFM)

**Figure 6:**
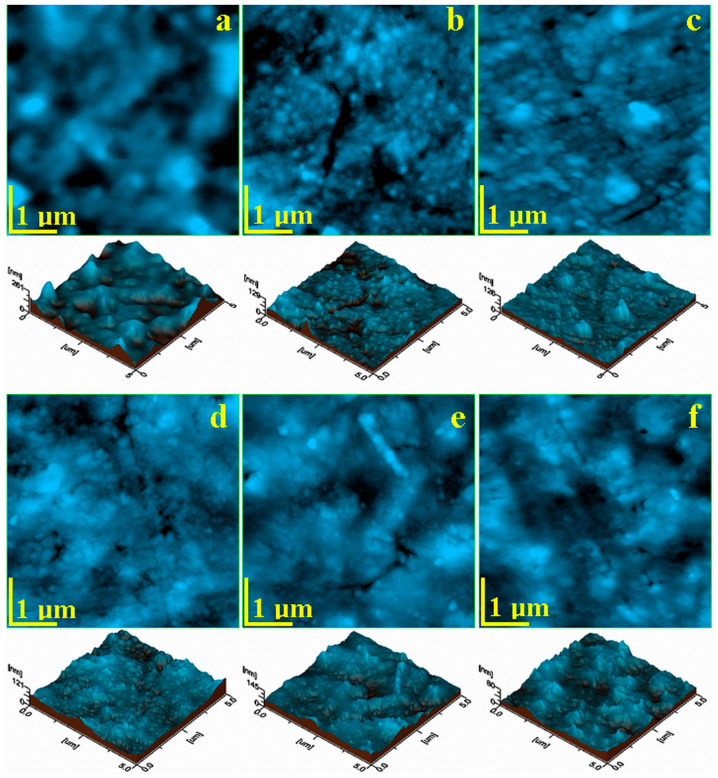
AFM images of the samples’ fine microstructures before liquid immersion: (**a**) S0, (**b**) S1, (**c**) S2 and after liquid immersion for 28 days: (**d**) S0, (**e**) S1, and (**f**) S2.

The SEM investigation of the sample surfaces reveals a compact and smooth surface for all the composite samples and mild infiltration between exposed filler particles and the polymer matrix, causing local mineral loss that was limited to the outermost layers. Therefore, a close observation of the fine microstructural details is required. This aim is fulfilled by the AFM investigation at scan size of 20 μm × 20 μm, shown in Figure 6.

The topography of the S0 sample, show in Figure 6a, revealed significant amount of glass particles with boulder-like shapes, having irregular margins. This aspect was most likely induced by the advanced milling of glass particles to fit the desired size range of 5–7 μm. Rounded particles of about 1–3 μm were also found, which belong to the quartz fractions of the mineral filler. These were very well-embedded into the polymer matrix, which contained a very well-dispersed nano hydroxyapatite. Larger particles were found on the sample surface, as observed in the three-dimensional profile presented below in Figure 6a. This induced a relatively high value of the roughness, which will be statistically discussed.

Sample S1 presented a more compact surface that better embedded the mineral filler in a more refined manner, shown in Figure 5b. Most of the larger glass and quartz particles were kept in the deeper layers, and only a few particles reached the outermost layers, as observed in the left side of Figure 6b. This induced small topographical irregularities that influenced the roughness values. The graphene oxide presence in the mixture facilitated better mixing of the smaller filler particles, assuring better cohesion. It was sustained by the topography of the S2 sample (which had the greater amount of graphene oxide), shown in Figure 6c, which had a smooth, compact, and dense fine microstructure. Only a few superficial submicron depressions occurred, which were about 500–800 nm, and they did not penetrate the deeper layers; therefore, we cannot categorize them as pores.

The liquid exposure of the S0 sample induced severe topographical changes, as observed in Figure 6d. All of the larger filler particles such as glass and quartz were affected by the liquid penetration between their surface and polymer matrix, causing their progressive delamination and subsequent mineral loss. This was sustained by their empty places forming superficial pores like the one situated in the middle right side of Figure 6d, which had the same irregular shape and size like the glass particles that were lost. The larger particles loss from the composite surface induced a significant decrease in the surface roughness.

The topography of the S1 sample after 28 days of liquid immersion revealed a relatively uniform aspect, which had some superficial pores induced by the outermost layer of glass particles loss (Figure 6e). This was also caused by the liquid infiltration between the particles and polymer matrix, causing their delamination.

Smaller filler fractions were also subjected to the liquid action, causing their removal and clustering as erosion debris found randomly on the surface like the ones observed in the lower side of the topographic image in Figure 6e. This can be better observed in the three-dimensional profile presented below the topographic image, causing a significant roughness increase.

The liquid exposure of the S2 sample had a minimal effect on the surface. Some small mineral particles dislocated due to their direct wetting and subsequent loss, shown in Figure 6f. Some small pores of 500–1000 nm were observed on the surface but did not penetrate into the deeper layers. Thus, the surface roughness had a mild increase. This proves that the graphene oxide presence brings microstructural cohesion, explaining the superior behavior of the sample compared to the behavior after 28 days of wetting.

**Figure 7:**
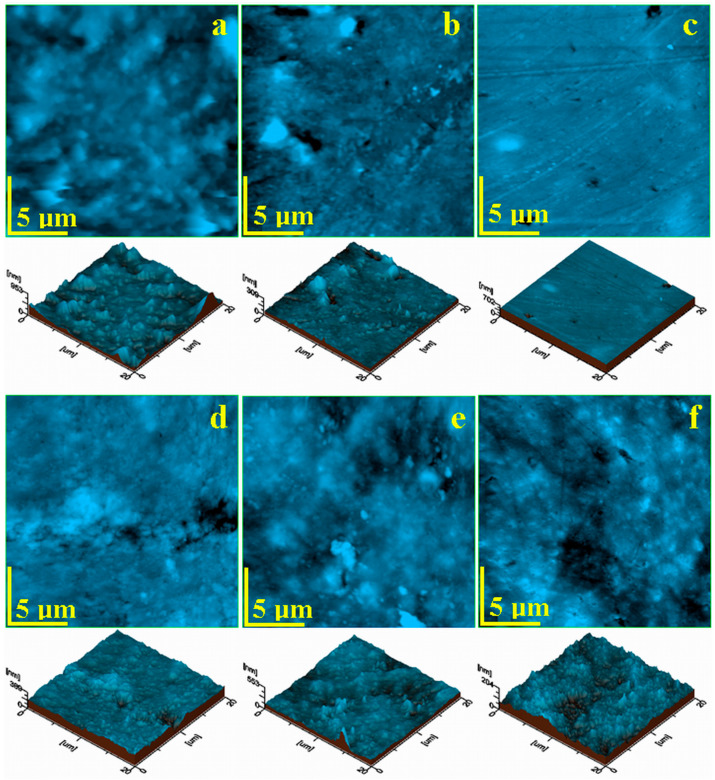
AFM images of the sample’s nanostructure before liquid immersion: (**a**) S0, (**b**) S1, (**c**) S2 and after liquid immersion for 28 days: (**d**) S0, (**e**) S1, (**f**) S2.

The fine microstructure observation does not allow for proper observation of the ultrastructural details. Therefore, the samples surface required a more detailed investigation at a scanned area of 5 µm × 5 µm, shown in Figure 7.

The topography of the unexposed S0 at an ultrastructural level, shown Figure 7a, revealed that the finest quartz particles were well-embedded into the polymer matrix containing nano hydroxy-apatite particles that were not visible due to the close packaging of the structure and a local height of 261 nm (as observed in the 3d profile of the topographic image presented below in Figure 7a). The filler particle disposal under the S0 ultrastructure implies a relative high value of the roughness.

The graphene oxide addition in S1 sample acted as an ultrastructural mediator be-tween nano hydroxyapatite particles and the polymer matrix, enhancing their distribution. The large agglomerations were avoided, generating a dense and well-structured network of submicron clusters shown in Figure 7b. These brought consistency to the material, which was observed based on enhancements in the mechanical properties. Their shape was spherical, and their diameter ranged from about 150–300 nm. Freely dispersed hydroxyapatite nanoparticles were present in the mixture as very small dots surrounding the submicron clusters. Better refining of the S1 ultrastructure induced by the presence of graphene oxide implies significantly lower roughness.

The ultrastructure of the S2 sample was more compact and denser, as observed in Figure 7c. The ultrastructural submicron clusters were also rounded, with diameters ranging from about 200 to 500 nm. They were very well-embedded into the polymer matrix, containing freely dispersed hydroxyapatite nanoparticles which were not visible due to the sample’s compactness. Thus, the S2 surface roughness at the nanostructural level was comparable with S1.

The prolonged exposure of the composites to the liquid also affected the nanostructural level. The quartz particles within the S0 sample surface were completely lost as a consequence of the liquid infiltration between their surface and polymer matrix, causing a massive delamination with significant mineral loss. Figure 7d evidence the empty places where quartz particles were situated on the outermost layer, forming pores of their shape and size (e.g., rounded shape of about 1 µm in diameter). The massive erosion of the larger feature allowed for the nanostructure observation: fine hydroxyapatite nanoparticles of about 60 nm were observed as being well-embedded into the matrix and forming some randomly disposed submicron clusters in the range of 150–500 nm.

The S1 sample after water exposure, shown in Figure 7e, revealed a topography affected by the local loss of larger features, forming pores of about 1 µm in diameter at the delamination sites. The hydroxyapatite submicron clusters were partly affected, generating the erosion debris observed in Figure 6e. The partial erosion at the nanostructural level of the S1 surface allows for the observation of the well-individualized hydroxyapatite nanoparticles of about 60 nm. The local alterations in the nanostructure caused a significant roughness increase.

Similar behavior was observed for the S2 sample shown in Figure 7f. The larger filler particles were lost due to their delamination, and the nanostructural features were partly eroded, forming some local submicron deposits and avoiding their coalescence onto larger dirt clusters, as occurred in S1 sample. Thus, the surface roughness iwas also significantly increased.

**Figure 8:**
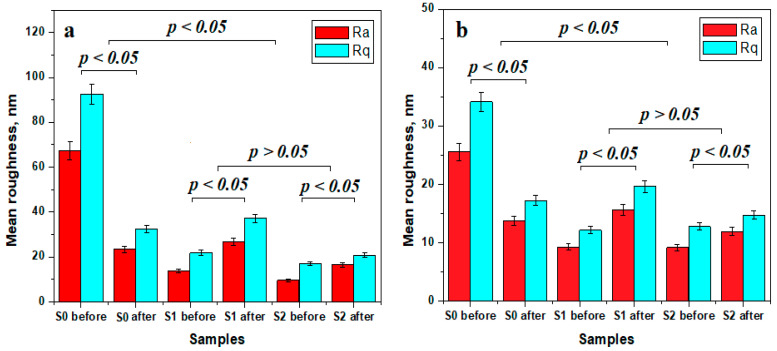
Surface roughness variation for the (**a**) fine microstructure and (**b**) nanostructure.

The roughness variation within all the investigated samples was statistically analyzed and displayed in Figure 8. Significant statistical differences were observed (*p* < 0.05) between the initial state of the sample and after exposure to water. The roughness significantly decreased for S0, both at the fine microstructure and nanostructure levels, while for S1 and S2, a significant statistical increase in the surface roughness was observed. Both the obtained values and their variations indicated a relevant statistical group formed by the S1 and S2 samples (*p* > 0.05), while S0 represented a different statistical group due to both values and their variation tendencies.

### 3.6. Antibacterial Test

After the end of the incubation period at 25 °C, the zones of inhibition (mm) were determined for the tested microbial strains. It was observed that against all the bacterial strains studied, the tested samples showed a good inhibition but varied in the size of the diameter depending on the tested microbial strain (Table 1).

**Figure 9:**
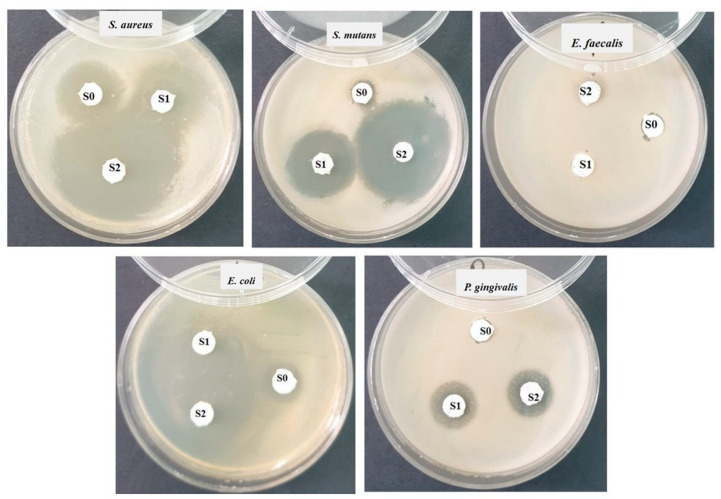
Antibacterial activity of the cement samples (S0, S1, S2), after 48 h of incubation against Sa—*Staphylococcus aureus*, Ec—*Escherichia coli*, Ef—*Enterococcus faecalis*, Pg—*Porphyromonas gingivalis*, Sm—*Streptococcus mutans*.

Figure 9 is correlated with Table 3 and allows for the visualization of the diameters of the bacterial inhibition zones of cement samples S0, S1, and S2, against the five types of bacterial strains.

The best antimicrobial activity was observed against the Gram-positive bacterial strain *Staphylococcus aureus* ATCC 25923. All the tested samples had a strong inhibition of staphylococcus, but the S2 sample reached an inhibition diameter of 27 ± 2 mm (Figure 9). Comparing the results, there were significant differences between S1 and S3 and S1 and S2.

Against the Gram-positive bacterial strain *Streptococcus mutans* ATCC 25175, very good inhibition was observed for samples S1 (19 ± 2 mm) and S2 (26 ± 2 mm), but sample S0 showed a very low inhibition (7 ± 2 mm) (Figure 9). In this case, there were statistical differences between all three samples investigated.

No bacterial inhibition was recorded after 48 h of incubation at 25 °C (Figure 9) against the Gram-positive bacterial strain *Enterococcus faecalis* ATCC 29212.

Good antimicrobial activity was observed against the Gram-negative bacterium *Escherichia coli* ATCC 25922. All the tested samples had inhibition of the bacillus, but sample S2 reached an inhibition diameter of 25 ± 2 mm. Comparing the results, there were significant differences between S1 and S3 and S1 and S2.

Sample S0, which did not contain GO but contained a maximum percentage of HA-Ag, did not register any antibacterial activity against the Gram-negative strain *Porphyromonas gingivalis* ATCC 33277. However, samples S1 and S2 showed a diameter of the inhibition zone of 12.5 ± 1 mm. It can be seen that a combination of the two powders (GO and HA-Ag) in the composition of samples S1 and S2 helped improve the antibacterial effect. For these bacterial strains, samples S2 and S3 showed statistical differences compared to S0.

According to the statistical tests, there were no significant differences regarding the absorption of water over time or changes in the surfaces of the investigated samples. In-stead, the antibacterial effect and mechanical resistance increased with increases in the GO%wt.

## 4. Discussion

In general, the inorganic filler in the resin matrix cements ranges from 60 to 75 wt%. The inorganic filler contains particles of different shapes and sizes, being composed of barium fluoro alumino borosilicate glass powders, strontium, zirconium, quartz, amorphous silica, and others [23].

The silanization of the inorganic filler helps to form the strong chemical bonds (of siloxane and hydrogen) between the resin matrix and the filler particles that contain silicon, such as quartz, SiO_2_, and silicate glasses, improving the interfacial bond between them [24]. Silanes have the role of coupling agents, being also called “molecular bridges”, helping to significantly improve the physical–mechanical, electrical, thermal, and optical properties of the composites [25].

Data from the literature show that by adding GO powder functionalized with silane, the adhesion and mechanical properties of the resin composites are stronger due to the presence of hydroxyl and epoxy groups on the basal plane and carbonyl and carboxyl groups on the edge of the GO sheets, which are able to form covalent bonds with the resin matrix [5]. Thus, the silanized GO sheets can provide an elastic layer that is capable of absorbing shocks and distributing stress more evenly throughout the material [16], improving its rigidity. Medeiros et al. think that the HA nanoparticles could have some negative impact on the mechanical properties, having a tendency to agglomerate. Areas with agglomerates can lead to defects in the structure of the material, causing cracking during mechanical testing and acting as stress points in the resin matrix. By introducing graphene oxide (0.05–0.3% *w*/*w*) next to HA (20% *w*/*w*), they tried to improve the mechanical and surface properties of the material. Increasing the addition of GO decreases the negative effects of HA, but the addition of GO must be very small to obtain an improvement in the mechanical properties [26]. The addition of GO therefore helps the distribution of nanoparticles in the matrix but also has a bioactive role in dental cements [18].

The conversion degree of the resinous composites refers to the non-reacted methacrylic groups after polymerization compared to the methacrylic groups in the non-polymerized material, indicating how the polymerization proceeded. An efficient polymerization can have beneficial effects on the physical–mechanical properties, absorption, and solubility of the materials. A DC between 60 and 75% was considered clinically acceptable [27]. A series of parameters such as polymerization mode, exposure time, or distance, can influence the polymerization of resin matrix cements. DC percentages between 55 and 75% have been reported for dual-cured resin matrix cements. The DC percentage of two known resin cements with dual polymerization, RelyX ARCTM (3M Svenska AB, Sollentuna, Sweden) and Variolink IITM (Ivoclar Vivadent GmbH, Ellwangen, Germany), was 72.8% and 65.7%, respectively. The degree of conversion can be improved by modifying the mentioned parameters [28]. In the case of our study, the thickness of the hardened sample was 1 mm, and the polymerization was carried out for 20 s on both sides. After 24 h, the DC% increased for the cement samples (over 70%), which indicates that the polymerization was efficient. An addition of 0.1% GO was sufficient to obtain a high DC% (86.2%). It was also observed that the inorganic filler improved the DC of all the investigated cements.

Bis-GMA combined with TEGDMA monomer (with a lower viscosity) improves the conversion degree and loadings capacity with filler but decreases the mechanical properties, increasing the polymerization shrinkage and water sorption [29]. Regarding to the flexural strength, it has been shown that it is more sensitive to the physical cross-linking density and not to the chemical one. In the case of an organic matrix based on Bis-GMA/TEGDMA, it was demonstrated that with the increase in TEGDMA addition, the FS decreases due to the decrease in the physical crosslinking density, although the chemical crosslinking density increases. Also, a content of 40 wt.% of TEGDMA in composites based on Bis-GMA/TEGDMA with silanized glass fillers had the positive effect of increasing DC and crosslinking density. Higher additions of TEGDMA led to reductions in the elastic modulus due to the decrease in the physical density of crosslinking and the concentration of rigid aromatic structures [30].

In the case of our study, the decrease in DTS for the S2 material can be explained by the agglomeration of the GO sheets in certain areas inside the samples, where stress can accumulate that can reduce the material’s resistance.

Water sorption (WS) can lead over time to the degradation of the cement through microleakages that can initiate the development of secondary caries, leading to pulpal inflammation and to fracture of the restoration through hygroscopic expansion. WS can also affect the mechanical properties of dental cements, causing occlusal changes. Therefore, a rigorous testing of the materials is necessary from this point of view as well. The factors that can affect the WS of cements include the hydrophilicity of the monomers in the polymer matrix, their degree of reticulation, the degree of conversion, the concentration and nature of the powders used as inorganic fillers, or the nature of the environment they come into contact with. The polarity of the molecules and the presence of unsaturated bonds between the polymer molecules can influence the WS, leading to the appearance of irreversible defects in the polymer matrix due to the damage of the filler/matrix interface [31,32]. The hygroscopic expansion caused by WS can also have beneficial effects by regulating the marginal adaptation, compensating for the initial contraction of the polymerization. Air bubbles on the surface due to manual mixing can partially inhibit the polymerization of the resin, leading to an increase in the residual monomer and its solubility. Also, the pores on the surface of the material favor the infiltration of the fluid through the cement, leading to swelling of the surface. Therefore, the negative aspect of large WS is that it can become the main cause of fractures [33]. It was found that the lower the crosslinking density, the higher the WS, and the higher the number of ethylene oxide sequences in a chain (especially more than three), the higher the WS [30]. The cement samples investigated by us registered values of WS until the end of the investigation period of below 40µg/mm^3^ which is the upper limit of WS for dental restorative materials [20].

To avoid the deposition of bacterial plaque, the surface of the restoration should ideally be smooth. The differences in roughness of different resin-based restorative materials are due to the size of the filling particles. For example, Fuji II LC (with particle dimensions of 5.9 μm) has a higher roughness than Filtek Z550 (0.02 μm). In addition to the size of the particles, their shape, their quantity, and their distribution, the type of polymer matrix can influence the roughness of the surface. The roughness, together with the surface integrity, can be associated with the dental restoration itself because it can negatively affect the marginal integrity, leading to the accumulation of bacterial plaque, followed by gingival irritation and then to clinical failure [34]. The experimental cement sample with GO 0.2% (S2) had a slightly increased roughness following the total period of its storage in water, but the surface roughness of S2 at the nanostructural level was comparable to sample S1 (with 0.1% GO). Polishing of restoration involves smoothening the surface, leading to the lowest surface roughness, minimizing the plaque accumulation on surfaces of restorations and minimizing the risk of bacterial colonialization [35].

The AFM investigation showed that the addition of GO enhanced the micro- and nano-structural properties of the composite samples, ensuring better bonding of the filler particles within the polymeric matrix. Thus, the initial surfaces of S1 and S2 were very compact and smooth, and after the liquid exposure, only the smaller filler particles directly exposed to the liquid within the outermost layers were delaminated and lost. Consequently, the surface roughness (both Ra and Rq) had a moderate but significant increase. Conversely, S0 (without GO) had a completely opposite behavior, with high roughness values in the initial state due to the bigger filler particles exposed on the outermost layer. Their delamination and loss due to the effects of liquid exposure caused a significant roughness decrease in the S0 sample. Therefore, GO addition ensures a better bonding of the bigger filler particles in the composite bulk, avoiding their exposure on the outermost layers and preventing the superficial damages caused by the prolonged liquid exposure.

The antibacterial effects can be due to the edges with sharp corners of the graphene sheets, which can cause damage to the cell membrane wall when in contact with bacteria [15]. At the same time, graphene can also act as an electron acceptor, being able to extract electrons from the bacterial membrane and compromising its integrity. ROS generation by GO can lead to oxidative stress that can cause mitochondrial dysfunction through DNA damage, which then leads to bacterial inhibition [36]. The presence of HA-Ag silver, together with GO powder, can create a synergistic effect for inducing a potential antibacterial effect of the investigated cements. However, our study shows that with the increase in the content of GO and with the respective decrease in the content of HA-Ag, a more pronounced antibacterial effect was obtained, although the two additions varied very little.

The present study presents a series of limitations. One of these limitations is related to the storage environment of the samples (distilled water), because in the oral environment the pH is different depending on the diet, affecting the absorption/solubility, roughness of the surfaces, damage to the surfaces, and the mechanical resistance. Also, testing the antibacterial effect can be extended to several types of bacteria that are constantly encountered in the oral environment. However, the experimental cements S1 and S2 demonstrated important potential and can be proposed as posterior restoration materials.

## 5. Conclusions

Three dental cements with and without graphene oxide (GO) were prepared and investigated. After 24 h, all the cements had a DC of more than 70%, with the highest value for S1 of 86.2%. The mechanical properties except for DTS improved with higher GO additions compared to the sample with only HA-Ag. The DTS values were higher for the S2 sample, and the YM values were higher for the S1 sample. After 28 days of storage in distilled water, the WS values increased with the percentage of GO but remained well below the ISO 4049 standard limit. SEM and AFM revealed no surface changes after 28 days in water. The antibacterial effects varied depending on the samples and bacterial strains tested, with inhibition zone diameters between 7 and 27 mm. Increasing the %wt GO and decreasing the %wt HA-Ag led to a more pronounced antibacterial effect.

The presence of GO, even in very low percentages, improved the properties of the experimental cements tested.

## Figures and Tables

**Table 1:** The composition of the experimental composite materials.

Composite Code	Organic Matrix	Inorganic Filler/Organic Matrix Ratio	Inorganic Filler wt.%				
			GO	La-Zr Glass	Sr-Zr Glass	HA-Ag	Quartz
S0	^1^ Bis-GMA ^2^ TEGDMA ^3^ Cq ^4^ DMAEM	70/30	0	35	5	10	50
S1			0.1	35	5	9.9	50
S2			0.2	35	5	9.8	50

^1^ Bis-GMA: Bis-GMA2 (mixture of higher oligomers: 81% monomers, 17% dimers, and 1% trimers): the base monomer, synthesized in the Polymeric Composites Laboratory of UBB-ICCRR; ^2^ TEGDMA: Triethylene glycol dimethacrylate (Sigma-Aldrich Chemie GmbH, Steinheim, Germany); ^3^ Cq: Camphorquinone (Sigma-Aldrich Chemie GmbH, Steinheim, Germany); ^4^ DMAEM: Dimethyl aminoethyl methacrylate (Sigma-Aldrich Chemie GmbH, Steinheim, Germany).

**Table 2:** The FS, CS, DTS, and YM of cement samples along with their standard deviations (SDs) (values with the same letters do not show statistical differences based on the ANOVA).

Samples	CS ± SD [MPa]	DTS ± SD [MPa]	FS ± SD [MPa]	Young’s Modulus ± SD [GPa]
S0	136.16 ± 10.83 ^a^	161.00 ± 20.86 ^a^	109.62 ± 6.43 ^b^	32.41 ± 6.84 ^a^
S1	136.21 ± 21.92 ^a^	162.32 ± 19.08 ^a^	118.57 ± 10.23 ^a^	29.52 ± 3.34 ^b^
S2	157.40 ± 18.00 ^b^	141.31 ± 20.01 ^b^	121.71 ± 11.67 ^a^	33.78 ± 6.24 ^a^

**Table 3:** The diameters of the inhibition zones (mm) of the tested samples.

Bacterial Strains	Sample 1 (S0) ± SD	Sample 2 (S1) ± SD	Sample 3 (S2) ± SD
*Staphylococcus aureus* ATCC 25923	15 ± 1	17 ± 2	27 ± 2
*Enterococcus faecalis* ATCC 29212	0	0	0
*Escherichia coli* ATCC 25922	10 ± 0.5	12 ± 1	25 ± 2
*Streptococcus mutans* ATCC 25175	7 ± 2	19 ± 2	26 ± 2
*Porphyromonas gingivalis* ATCC 33277	0	12.5 ± 1	12.5 ± 1

## Data Availability

The original contributions presented in the study are included in the article, further inquiries can be directed to the corresponding author.
